# Four Steps To My Future (4STMF): acceptability, feasibility and exploratory outcomes of a universal school‐based mental health and well‐being programme, delivered to young adolescents in South Africa

**DOI:** 10.1111/camh.12660

**Published:** 2023-07-13

**Authors:** Bronwynè Coetzee, Maria Loades, Suzanne Human, Hermine Gericke, Gerrit Laning, Martin Kidd, Paul Stallard

**Affiliations:** ^1^ Department of Psychology Stellenbosch University Stellenbosch South Africa; ^2^ Department of Psychology University of Bath Bath UK; ^3^ Community Keepers, Non‐Profit Company Stellenbosch South Africa; ^4^ Department of Statistics and Actuarial Sciences Stellenbosch University Stellenbosch South Africa; ^5^ Department of Health University of Bath Bath UK

**Keywords:** Mental health, young adolescents, universal intervention, school‐based, LMIC, South Africa

## Abstract

**Objective:**

Mental health disorders affect many children in South Africa, where vulnerability is high, and treatment is limited. We sought to determine the feasibility and acceptability of a universally delivered classroom‐based programme for the promotion of mental health in young adolescents.

**Method:**

We pilot tested an 8 session, cognitive‐behavioural therapy‐based programme, 4 Steps To My Future (4STMF) in two schools. Participants were grade 5 learners (*n* = 222; Mean_age_ = 10.62 (Standard deviation = 0.69)). 4STMF was delivered in class time by trained psychology postgraduates. Feasibility (rates of parental opt‐out, child assent, assessment completion at baseline and follow‐up, programme completion, session attendance and programme fidelity), acceptability (teacher feedback and focus groups with learners), as well as demographic data and data on a battery of a psychological measures were collected at baseline, postintervention and at one‐month follow‐up.

**Results:**

Most eligible learners at both schools agreed to participate (85% – school 1; 91% – school 2) with more than 80% completing postintervention measures. Learner session attendance and programme fidelity were high. Teachers rated facilitators highly on confidence, preparedness, enthusiasm and classroom management and observed children to be enjoying the programme. Focus group data suggest that learners liked the programme, could recall the content and had shared some of the content with their family. An exploratory analysis of outcomes showed significant pre–post differences on self‐esteem at school 1 and on emotion regulation at school 1 and school 2, maintained at follow‐up.

**Conclusions:**

This pilot study has shown that 4STMF can acceptably and feasibly be delivered, at classroom level, as a universal school‐based prevention programme to young adolescent learners in South African primary schools. The programme could fit in with school context, could be delivered by nonspecialists, showed significant improvements on self‐esteem and emotion regulation and was liked by the learners.

**Key Practitioner Message**

**What is known?**

- Common mental disorders like anxiety and depression are preventable through early intervention.
- Universal school‐based prevention interventions for mental health have been delivered to good effect in high‐income settings, but comparatively less is known about this in low‐ and middle‐income settings like South Africa.

**What is new?**

- We have demonstrated that it is feasible and acceptable to deliver a universal school‐based prevention programme at classroom level to young adolescents in an LMIC setting.
- We have also shown that the programme can be flexibly delivered.

**What is significant for clinical practice?**

- Delivery of programmes aimed at prevention of mental health conditions is possible and can be done at school level among young adolescents.
- Delivery of these programmes can be done by nonspecialists, which may alleviate the burden of patient load on public health care facilities.

## Introduction

Psychological distress, including anxiety and depression, is a pressing public health concern; the World Health Organisation (WHO) has identified improving mental health among young people as a key priority required to promote sustained economic and social development (World Health Organization, 2022). Psychological distress particularly increases during the teenage years, with an estimated 9% of young adolescents and 15% of older adolescents screening positive for an emotional disorder (NHS Digital, 2021). Of these, 2% of young people meet the criteria for a probable depressive disorder and more than 7% a probable anxiety disorder (NHS Digital, 2021). Many more will have subthreshold symptoms. Having mental health problems like depression in childhood and adolescence is associated with numerous unfavourable outcomes, both short‐term (Benarous et al., 2019; Finning et al., 2019; Schwartz‐Mette, Shankman, Dueweke, Borowski, & Rose, 2020; Wickersham et al., 2021) and into adulthood (Clayborne, Varin, & Colman, 2019; Copeland et al., 2013; Johnson, Dupuis, Piche, Clayborne, & Colman, 2018; Naicker, Galambos, Zeng, Senthilselvan, & Colman, 2013).

What makes the global burden of mental health problems among children and young people (CYP) even greater is limited access to timely, affordable and specialist treatment. In low‐ and middle‐income countries (LMICs) where vulnerability factors for developing mental health problems are even more pronounced (Thapar, Collishaw, Pine, & Thapar, 2012), and treatment provision is particularly stark (Flisher et al., 2012), scalable and culturally sensitive prevention and early intervention programmes with the potential to mitigate the onset of these disorders is an important public health priority (World Health Organization, 2022).

In South Africa, there is a considerable burden of mental health problems and lack of access to treatment. Notably, there is no robust national data available on the prevalence of common mental disorders in CYP (Herman et al., 2009), although smaller scale studies have pointed towards high rates of both anxiety and depression (Kleintjes et al., 2006; Muris, du Plessis, & Loxton, 2008). These high rates of anxiety and depression are not surprising, given exposure to high rates of violence, trauma and substance abuse, which increase the risk of poor mental health (Naicker, Norris, & Richter, 2021; Stansfeld et al., 2017). Qualitative work in the Western Cape specifically has elucidated from both CYP and parent perspectives the extent to which trauma is experienced in these communities and the need for professional support (Hiller et al., 2017; Williamson et al., 2017). Yet, there are numerous barriers to seeking psychological help, including stigma, financial cost, time and lack of confidence in the treatments offered (Mokomane, Mokhele, Mathews, & Makoae, 2017), and specialist treatment is scarce with estimates of one mental health professional per 5000 CYP (Flisher et al., 2012). Finding appropriate as well as cost‐effective ways to address these barriers have been a core focus for successful implementation and scale up of psychosocial interventions across a range of mental health conditions in low‐ and middle‐income countries (Asher, Patel, & De Silva, 2017; Bird et al., 2022). Indeed, a focus on nonspecialist delivery of mental health services and programmes in community‐based settings, and through participatory community‐based approaches have been key in establishing contextual fit and programme buy‐in from key stakeholders (Barnett, Gonzalez, Miranda, Chavira, & Lau, 2018).

National school attendance in South Africa was at 98% in 2020 for children ages 7–17 years (Hall, 2022; Statistics South Africa [StatsSA], 2020), and 94.6% in the Western Cape, making schools an ideal site for widespread school‐based prevention and early intervention efforts. Schools offer a particularly convenient setting for delivering universal, nonselective and widespread programmes aimed at building life skills and promoting mental health and well‐being. Internationally, there is a vast and rapidly growing literature reporting on school‐based interventions targeted at mental illness prevention and early intervention. Such interventions may either be delivered universally (to whole classes of learners, grades or schools), or to selected learners as targeted (i.e. individuals with some mental health problem symptoms) or indicated (i.e. individuals who are at particular risk of developing mental health problems) interventions. Evidence of effectiveness for universally delivered programmes is mixed, with better evidence of effectiveness for selective programmes (Kambara & Kira, 2021; Werner‐Seidler, Perry, Calear, Newby, & Christensen, 2017).

However, few universally delivered school‐based programmes have been developed and tested in LMICs. A systematic review of universal school‐based mental health programmes in LMICs conducted in 2019 found only 12 studies conducted in 11 countries, with none in South Africa. It is promising that studies reported improvements in depression and/or anxiety but few studies detailed how the interventions were developed or adapted to fit the local context (Bradshaw et al., 2021). Indeed, the design and adaptation of effective preventive interventions requires community ownership, cultural flexibility and fit with the delivery context to maximise effectiveness, appropriate training and support to deliver and relevance and acceptability to stakeholders (Kieling et al., 2011). While evidence from middle‐ and high‐income countries suggests that selective programmes are more effective, the limited resources available in LMICs suggests that universal programmes may offer a more pragmatic solution. Particular advantages of universal interventions are that they do not require rigorous screening and selection of participants, which in a school context can be stigmatizing and discourage participation. Universal interventions may also fit better within existing school timetables, given that they are, by definition, delivered to all learners, rather than to some and not others.

When developing interventions, evaluating implementation in practice is key to developing a scalable and sustainable solution that fits the specific context (Skivington et al., 2021). Implementation science is the study of methods, strategies and tools to promote the systematic uptake of research findings and other evidence‐based practices into routine practice in healthcare settings (Williams & Beidas, 2019). It involves understanding the factors that influence the adoption, implementation and sustainability of evidence‐based interventions, and developing strategies to facilitate their effective implementation in real‐world contexts. In the context of LMICs, implementation science is particularly important because these settings often face unique challenges (e.g. limited resources, inadequate infrastructure, cultural and language barriers and political instability) in implementing evidence‐based interventions.

Our primary aim in this study was to explore the feasibility and acceptability of delivering a locally developed universal prevention programme, 4 Steps To My Future (4STMF), in South African primary schools. As part of exploring acceptability, we specifically sought to explore barriers and enablers to implementation in practice to inform future developments. We also undertook a pre–post exploratory analysis of our psychological measures to inform decision‐making about the primary outcome measure and power calculation for a subsequent randomized controlled trial (RCT).

## Methods

### Research design

We conducted a pilot feasibility and acceptability trial of the 4STMF programme at two primary schools in the Western Cape. We collected pre‐, post‐ and one‐month follow‐up data at both schools. We also collected qualitative data from participants at both schools at one‐month follow‐up. The study protocol was published (Coetzee, Loades, et al., 2022) and the trial was preregistered (Pan African Clinical Trial Registry PACTR202004803366609).

### Setting and participants

In South Africa, learners attend primary schools from Grade 1 (approximately age 7) to Grade 7 (approximately age 14). The two participating public schools were randomly chosen from a list of 21 primary schools in the Boland area of the Western Cape who receive psychosocial support services from a local Non‐Governmental Organisation (NGO) partner, Community Keepers. Both schools were eligible for the National School Feeding Scheme (Devereux et al., 2018), which supports those schools where learners are predominantly from low socioeconomic backgrounds. Typical class sizes are 40 learners per class, although during COVID‐19 related restrictions class sizes were halved to accommodate social distancing. All grade 5 learners (6 classes school 1; 8 classes school 2) were invited to take part in this study.

### Description of the intervention

4 Steps To My Future (4STMF) is a bespoke, locally developed programme, informed by cognitive‐behavioural therapy (CBT). It focuses on developing four core skills of enhancing self‐esteem, promoting helpful thinking, developing emotional regulation and problem solving/goal planning. For more details on the content of 4STMF, see study protocol (Coetzee, Loades, et al., 2022). The programme required just over 3 hr for delivery, with each core skill focused upon during two, 20–25 min sessions, the length of a typical school lesson. Two trained psychology postgraduate students delivered the intervention to each class, with a third acting as an intervention observer and supporter. The facilitators followed a manualized programme (available from the corresponding author on request), and learners completed worksheets related to the content of each session. Posters summarizing key points were hung on classroom walls after each step. Learners were also handed information leaflets to take home to their parents at the end of each of the 4 steps summarizing the key points and ideas of how parents can support the steps in the home. For details of how 4STMF was developed, and how the facilitators were trained and supervised, see study protocol (Coetzee, Loades, et al., 2022).

For pragmatic reasons related to COVID‐19 restrictions and school‐based preferences, the 4STMF programme was delivered once a week to school 1 during Terms 2 and 3, May to August 2021. In school 2, 4STMF was delivered more intensively, four sessions per week (2 sessions per day, twice a week) during Term 3 (August to September 2021). School 2 requested that the intervention be delivered in this time frame, as they could not accommodate intervention delivery during scheduled examination periods.

### Measures

We determined feasibility, acceptability and exploratory outcomes of the intervention on several psychological measures.

#### Feasibility measures

We recorded rates of parental opt‐out, child assent, assessment completion at baseline and follow‐up, programme completion (number of groups who received all 8 sessions of 4STMF), session attendance (number of children who attended each programme session) and programme fidelity (i.e. number of sessions delivered fully, as intended). We used bespoke fidelity checklists to determine how confident, prepared and enthusiastic facilitators appeared while delivering the programme, as well as how well they managed behaviour in the classroom. We also used fidelity checklists to determine how long it took to deliver a session and whether each activity was delivered as intended. An observer (either a postgraduate psychology student, a school mental health counsellor or a senior member of the study) attended each session and completed this fidelity checklist. Fidelity checklists were also independently completed for each session by the lead facilitator and co‐facilitator.

#### Acceptability measures

The fidelity checklists included ratings of how much the children engaged with, participated in, enjoyed and understood each of the core tasks facilitated in that specific session of the intervention.

Postintervention, teachers were asked to provide written feedback about their impression of the facilitators, the intervention, how the children experienced the intervention, and how the implementation of the intervention might have impacted on their work responsibilities at school. Quantitative feedback consisted of teachers being asked to rate the facilitators on confidence, preparedness and enthusiasm (0 = not at all, 1 = a little, 2 = somewhat, 3 = quite a lot, 4 = a great deal). Teachers were also asked to rate learner participation, enjoyment and understanding of each lesson. Qualitative feedback was collected via a free text box asking teachers to write down any additional comments.

Qualitative, semistructured focus groups were conducted with CYP. Learners at both schools who received the intervention were invited to focus groups after the one‐month follow‐up data were collected at school 2 (week commencing 27 September 2021). Focus groups were conducted by a senior member of the research team who had not been involved in the delivery of the intervention.

#### Psychological measures

##### Sociodemographic variables

At baseline, participants reported their age, gender, first language, whether a school grade had ever been repeated, living situation, whether they had their own bedroom, number of people and rooms in the house and whether their home had a water tap inside.

##### Psychological measures

Participants completed the following psychological assessments at baseline, postintervention and one‐month follow‐up.

##### Anxiety and depression symptoms

We measured symptoms of anxiety and depression using the Revised Child Anxiety and Depression Scale‐30 (RCADS‐30) (Sandín et al., 2010). The RCADS‐30 asks respondents to rate each item on a scale of 0 ‘never’ to 3 ‘always’. Raw scores range between 0 and 90. Raw scores ≥49 are considered above clinical threshold (Stallard et al., 2014). The measure showed good reliability for the total sample at baseline (α = .84).

##### Self‐esteem

We measured self‐esteem using the Rosenberg self‐esteem scale (Rosenberg, 1965). This measure contains 10‐items, and respondents rate each item on a score of 1 ‘strongly agree’ to 4 ‘strongly disagree’. Items 3, 5, 8, 9 and 10 on this measure are reverse scored. The measure showed moderate reliability for the total sample at baseline (α = 0.59).

##### Goal setting

We measured goal setting using the Goal‐Setting Scale (Webb & Baer, 1995). The measure contains 5‐items. Responses are rated on a scale from 1 (‘I'm not at all like this and I don't believe it’) to 5 (‘This is exactly the way I am and what I believe’). Scores for items 4 and 5 are recoded so that higher scores indicate better goal setting skills. Reliability for this measure across the total sample at baseline was poor (α = .37).

##### Happiness and well‐being

We asked participants to rate their happiness about school, their appearance, family, friends, the home they live in, their health and life in general on a 7 item, visual analogue scale which had previously been used in a large UK trial with learners in schools (Stallard et al., 2014). The measure showed good reliability for the total sample at baseline (α = .67).

##### Emotion regulation

We measured emotion regulation using the 10‐item Emotion Regulation Questionnaire for Children and Adolescents (ERQ–CA; Gullone & Taffe, 2012). The ERQ–CA assesses the use of two different emotion regulation strategies, namely Cognitive Reappraisal Facet (CRF) and Expressive Suppression Facet (ESF). As such, a total score is not typically computed for this scale. The CRF subscale (α = .63) has 6 items (1, 3, 5, 7, 8 and 10) with a range of 6 to 30, and the ESF subscale (α = .59) has 4 items (2, 4, 6 and 9) with a range of 4 to 20. A score is calculated for each subscale, with higher scores indicating greater use of that particular emotion regulation strategy.

### Contextual measures

#### Bullying

We measured bullying using the 2‐item Olweus Bully/Victim Questionnaire‐Modified (Strohmeier et al., 2011). We asked children to indicate how often they have been bullied (been bullied), as well as how often they have taken part in bullying others (have bullied). Children have 5 responses to choose from for each question ranging from 1 ‘I haven't been or haven't bullied other children’ to 5 ‘I have been or have bullied other children several times a week’. Given these were only two‐items we did not calculate reliability.

### Procedure

All eligible grade 5 children were provided with an information sheet, which included an opt‐out consent form to be completed and returned to school should parents (caregivers) not want their children to take part. In addition to parent consent, written, assent was obtained from the children themselves after they had been given the opportunity to ask questions. Our parental opt‐out consent forms informed parents that their children may stay in the classrooms and participate in all activities related to 4STMF, but that their children will not be required to complete any assessments. We also indicted to parents that if necessary we could arrange for their children to be supervised in a separate classroom. We had no requests from parents to make such an arrangement.

All 4STMF sessions were delivered during class time. Teachers were not required to stay for the delivery of the sessions although their presence was encouraged to maintain discipline.

Postgraduate psychology students administered assessments during class lessons. Each child completed their own assessment booklet while a postgraduate psychology student read each question aloud. Other psychology students were in the class to provide assistance to those children who needed additional help.

At postintervention, children were invited to take part in a focus group to share their experience of the intervention. Interested children required parental consent and child assent to participate. The focus groups discussed the children's experience of the intervention, the facilitators and whether they preferred their teacher to be in the class or not. The focus groups were led by the principal investigator (BC) who was not directly involved in the delivery of the intervention. A total of 6 focus groups were conducted, 4 with school 1, and 2 with school 2. Focus groups ranged in size from 2 to 6 children per group with a total of 23 children (15 girls and 8 boys) participating.

### Ethics

We received permission to conduct this study from the Social, Behavioural and Education Research Ethics Committee (REC: SBER) at Stellenbosch University (project ID 9183) and reciprocity from the Psychology Research Ethics Committee, University of Bath.

### Data analyses

#### Quantitative data analysis

Data were entered into the Statistical Package for the Social Sciences (SPSS) version 28 for descriptive and inferential analysis. Data were analysed using SPSS v 28 and the R package for mixed model analysis of variance (ANOVA). All data were checked for normality.

Data on demographic and feasibility measures were analysed using descriptive statistics. We calculated means and standard deviations for continuous data, and frequencies and percentages for categorical data. For demographic data, we used Mann–Whitney U to compare schools on non‐normally distributed continuous data and Chi‐Square (*X* ^*2*^) to compare schools on categorial data. We considered differences between groups to be statistically significant at the 95% level.

Data on psychological measures were analysed using a two‐way mixed model ANOVA in R to calculate within group differences on measurement data at preintervention, postintervention and at one month follow‐up. We report p‐values as well as effect sizes (Cohens *d*) for the within group differences. Our data were not powered to detect significance and for this reason we are not reporting on between group differences. In R, the lmer() function was applied to deal with missing data via multiple imputation (MI).

#### Qualitative data analysis

Qualitative focus group data were transcribed verbatim in English and uploaded into ATLAS.ti V 8. We coded the data both inductively and deductively using thematic analysis (Braun & Clarke, 2006). The focus group data were read for familiarization, and we then created codes that captured participants' responses to the acceptability, understanding, relevance and helpfulness of the programme. Codes were closely related to specific questions we asked about what participants liked about the programme and the facilitators, what they understood and recalled about the programme and activities, whether the programme had a meaningful impact on their lives, whether they had told others about the programme, what influence having their teacher in class or not had, and in what ways the programme has helped them.

## Results

### Feasibility

**Figure 1:**
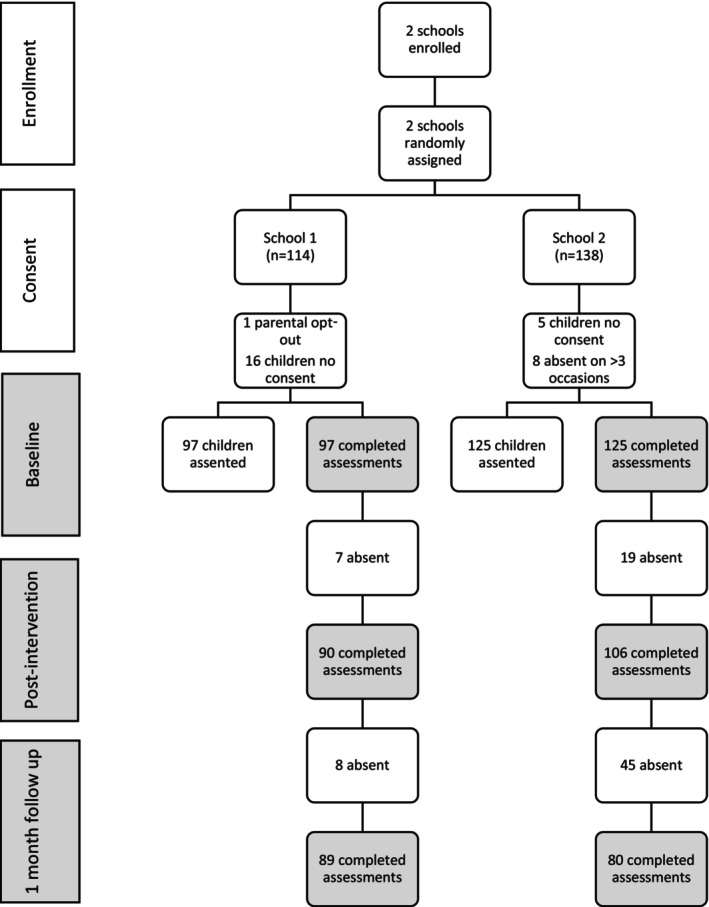
CONSORT diagram showing recruitment and retention of participants by group allocation

As can be seen in Table 1, school 1 had 6 grade 5 classes with 114 learners and school 2 had 8 classes with 138 learners. Of the 114 eligible learners in school 1, 97 (85.1%) provided consent and assent. Of the 138 eligible learners in school 2, 125/138 (90.6%) provided consent and assent. Learner attendance at each session was high at more than 76% attendance across both schools. Assessment completion rates were high at baseline and postintervention but dropped in school 2 at follow‐up. Figure 1 shows the consort diagram summarising participant flow at each time point.

**Table 1:** Feasibility outcomes of 4STMF session delivery across the two schools

	School 1	School 2
Number of children invited to take part at baseline	114	138
Rates of parental opt‐out	2 (1.75%)	0 (0%)
Rates of child assent (assent forms returned)	97 (85.1%)	125 (90.6%)
Rates of assessment completion (on all measures)		
Preintervention (baseline)	97 (100%)	125 (100%)
Postintervention	90 (92.78%)	106 (88.33%)
One‐month follow‐up	89 (91.75%)	80 (64.00%)
Rate of programme completion		
Number of grade 5 classes who received all 8 lessons of programme	6 (100.00%)	8 (100.00%)
Number of learners attending each lesson		
Lesson 1	93 (95.88%)	96 (76.8%)
Lesson 2	91 (93.81%)	96 (76.8%)
Lesson 3	84 (86.6%)	101 (80.8%)
Lesson 4	90 (92.78%)	101 (80.80%)
Lesson 5	91 (93.81%)	95 (76.00%)
Lesson 6	85 (87.63%)	95 (76.00%)
Lesson 7	91 (93.81%)	101 (80.80%)
Lesson 8	81 (83.51%)	101 (80.80%)
Programme fidelity (Sessions delivered fully as intended)	7/8 (87.50%)	7/8 (87.50%)

### Comparison of participant characteristics by school at baseline

The two groups of participants were comparable at both schools, with slightly more males and mostly Afrikaans speaking. Those at school 2 were older and more had repeated a grade. Learners mostly lived with both biological parents, had their own bedroom, had more than 2 people who lived at home with them and had access to a tap/faucet (see Table 2).

**Table 2:** Sample demographic characteristics by school at baseline

	School 1 (*n* = 97)		School 2 (*n* = 125)		*p*‐Value
	*M*/*n*	*SD*/%	*M*/*n*	*SD*/%	*U*/*X* ^2^
Sociodemographic variables					
Age (years)	10.46	0.54	10.74	0.76	**<.01**
Gender					
Male	58	59.80	65	52.00	.28
Female	39	40.20	60	48.00	
First Language (mother tongue)					
English	17	17.52	4	3.20	**<.01**
Afrikaans	72	74.22	114	91.20	
Other	8	8.25	7	5.60	
Any grade repeated ever (Yes)	8	8.25	37	29.60	**<.001**
Living situation					
Biological mother only	10	10.31	16	12.80	.07
Biological father only	1	1.03	3	2.40	
Both biological parents	43	44.33	33	26.40	
Other immediate relative	4	4.12	14	11.20	
Biological mother and partner	2	2.06	2	1.60	
Both grandparents	6	6.19	7	5.60	
Grandmother only	5	5.15	2	1.60	
Nonbiological caregiver	0	0	2	1.60	
Orphanage	0	0	2	1.60	
Other	26	26.80	40	32.00	
Own bedroom (yes)	58	59.79	66	52.80	.41
Number of people in household					
Mean (*SD*)	5.14	2.27	6.61	3.46	**<.01**
≥2	92	94.84	99	79.20	
≥10	5	5.15	26	20.80	
Number of rooms in house					
Mean (*SD*)	5.99	2.5	5.04	2.25	**<.01**
≥2	95	97.94	122	97.60	
Taps in house (Yes)	95	97.94	118	94.40	.30

The values in bold are the *p*‐values which are statistically significant at the 95% level.

### Acceptability

#### Findings from fidelity checklists

##### Observer feedback

Based on aggregated fidelity checklist data (see Table 3), a teacher was present in almost all sessions (82.5%) delivered at school 1 but in only 41.1% of the sessions delivered at school 2. Over 90% of sessions were delivered, as intended, by two facilitators. Average session attendance was 14–15 learners, with average session length ranging from 23 to 27 min. There was a higher occurrence of disciplinary actions and interruptions at school 2, with an average of 4 interruptions per lesson versus about 1 interruption per lesson at school 1. As can be seen from Table 3, facilitators at both schools were rated confident, prepared and enthusiastic. Learners at both schools were rated as engaged and participated in and enjoyed and understood the sessions.

**Table 3:** Summary of fidelity checklist data averaged across all eight sessions

	School 1	School 2
Educator present (% of the time)		
Yes	82.50	41.10
Some of the time	0.00	24.70
No	17.50	34.20
Both facilitators present (% of the time)	90.50	95.80
Language of instruction (%)		
Afrikaans	67.20	100.00
English	38.20	0.00
Participants per lesson		
Mean	15.03	14.22
*SD*	4.21	3.13
Session time (min)		
Mean	27:19:00	46:46:00
*SD*	03:31	06:52
Class observation (*M*, ±*SD*)		
Disciplinary issues	0.11 (0.45)	0.38 (0.83)
Adverse events	0.00 (0.00)	0.03 (0.17)
Interruptions	0.88 (1.14)	4.18 (4.34)
Facilitator assessment (*M*, ±*SD*)		
Confidence	3.34 (0.53)	3.58 (0.53)
Preparation	3.67 (0.49)	3.72 (0.45)
Enthusiasm	3.19 (0.41)	3.22 (0.55)
Management	3.65 (3.59)	3.59 (0.57)
Learner participation (*M*, ±*SD*)		
Engagement	3.04 (0.61)	2.99 (0.59)
Participation	3.20 (0.74)	3.11 (0.70)
Enjoyment	2.87 (0.71)	2.86 (0.71)
Understanding	3.32 (0.63)	3.47 (0.64)

##### Teacher feedback

At school 1, 4 (80%) of 5 teachers provided feedback, and at school 2, 1 (25%) of 4 did. Teachers across both schools rated facilitators as confident, prepared and enthusiastic (*M* = 4; *SD* = 0) and rated their classroom management highly (*M* = 3.75; *SD* = 0.5). One teacher commented that the facilitators' excitement about working with the learners made the learners excited about the lessons. Teachers perceived learners to engage with (*M* = 3.5; *SD* = 0.6), participate in and enjoy the lessons (*M* = 4; *SD* = 0), to have good understanding of the lessons (*M* = 3.75; *SD* = 0.5) and thought that the lessons had made a difference in the children's lives (*M* = 3.5; *SD* = 0.6). One teacher noted in her feedback that some of the learners, who are withdrawn, ‘came out of their shells’ while participating in the intervention. Another teacher noted that at times the learners did not have the ‘necessary vocabulary’ to take part in discussions, which then resulted in them being somewhat nervous to share their opinions. Teachers themselves rated the lessons as meeting their expectations and enabling them to learn something new (*M* = 4; *SD* = 0) and were enthusiastic about the programme (before implementation (*M* = 3.6; *SD* = 0.5) and postimplementation (*M* = 4; *SD* = 0)). While teachers rated the programme as adding somewhat to their workload (*M* = 1.75; *SD* = 2.1), they reported that it did not take away from the time to finish the school curriculum (*M* = 0.3; *SD* = 0.6). One commented that the programme was never seen as ‘extra work’ or as a burden, but as an opportunity for learners to be introduced to something new, an opportunity to focus on themselves and their feelings, which is not something that they as teachers always get to do with the learners.

##### Learner feedback

We analysed the focus group data collected after the delivery of the intervention to learners at both schools. Please see supplementary data for a table of themes and subthemes, with supporting quotations (see Table S1).

Learners enjoyed the programme and stated that the facilitators made them feel comfortable to express their emotions in a classroom setting. Learners said the programme helped them to feel better, and to think about their future. While most enjoyed activities like the breathing activity and the kindness activity, some mentioned feeling shy doing these in class. Learners suggested that the programme could be further improved by including more games, limiting the number of worksheets and by having more sessions. Participants were able to recall some of the content of various sessions and activities and remarked that the classroom posters created for each step were helpful reminders of these skills. Learners reported telling their parents, siblings and friends about what they had learned and handed the parent handouts sent home after each step to a parent or caregiver.

### Psychological measures: exploratory analysis

#### Within group differences at school 1

We found differences in mean scores at baseline, postintervention and one‐month follow‐up at school 1. There were no significant differences in pre–post scores on any measures except the Rosenberg self‐esteem scale (*p* < .01, effect size (ES) = 1.36 (large effect)) and the Emotion regulation questionnaire‐expressive suppression facet (*p* < .01, ES = 0.44 (medium effect)). This difference was maintained at follow‐up on both measures (see Table S2 here).

#### Within group differences at school 2

We found differences in mean scores at baseline, postintervention and one‐month follow‐up at school 2. There were no significant differences in pre–post scores on any measures except the Emotion regulation questionnaire‐expressive suppression facet (*p* < .01, ES = 0.57 (medium effect)). This difference was maintained at follow‐up (see Table S3 here).

## Discussion

In the context of the urgency with which to address the growing burden of mental health conditions among CYP (Holmes et al., 2018), our findings demonstrate that it is possible to deliver a universal, school‐based programme and that doing so fits with stakeholder expectations and needs, which is key for future implementation. LMICs, largely characterised by widespread poverty, stigma, social inequality, political instability and adversity, bear the burden of global mental health conditions, yet paradoxically in these settings there are fewer resources available to address mental health needs. Consequently, there has been much focus on finding cost‐effective, culturally sensitive and contextually appropriate ways to address these needs, and with a growing emphasis on prevention and early intervention. While there is a growing body of evidence demonstrating the success of delivering cost‐effective interventions delivered by nonspecialists through task‐shifting approaches in community‐based settings to address a range of mental health conditions (Barnett et al., 2018), there is considerably less known about universal and early interventions delivered to CYP in school‐based settings in LMICs and in South Africa (Bradshaw et al., 2021) and this work offers encouraging findings.

Based on this feasibility trial, conducted peri‐pandemic in 2 primary schools in South Africa, we found promising indications that it would be possible to run a fully powered trial of the 4STMF programme as a universal, CBT based intervention delivered to classes of grade 5 learners. In particular, we demonstrated that it is possible to recruit schools, recruit 85%–90% of eligible learners and deliver 4STMF in situ following the programme manual. We were also able to use questionnaire outcome measures and to retain participants at one‐month follow‐up, although the retention of only 64% of learners at school 2 needs to be enhanced. This may indicate a lower level of commitment to the programme in school 2 or might reflect other academic pressures and priorities within the school as students prepared for exams. Exploring these potential barriers to recruitment in consultation with schools and findings ways to address these will be important for a future trial and scale up. Encouragingly, the learners liked the programme, remembered activities related to the sessions and described ways in which they had used these outside of the sessions themselves, indicating both grasp and generalization of these skills. Facilitators, who were graduate students, were able to deliver the programme with relatively little training, making it potentially scalable in the future. These feasibility and acceptability findings demonstrate that 4STMF can feasibly be delivered in a community‐based setting, and delivered by nonspecialists addressing many of the known barriers to successful implementation of psychosocial interventions such as stigma, cost and lack of access to specialist services.

This study was not powered to examine the effect of the intervention although an exploratory analysis suggests that effect sizes on our outcome measures varied. This is typical of universal interventions where the majority of participants are relatively healthy and improvements are more modest (Cuijpers, 2022). Nonetheless, one of the advantages of universal programmes is the ability to enhance skills to maintain well‐being and to prevent future deterioration. Modest short‐term reductions may, therefore, be accompanied by longer‐term benefits and that is important to assess. In addition, we also note that the reliability of some of the measures we tested were poor and further attention needs to be paid to enhancing the understandability of some questionnaire items. These results will help us to consider our assessment time point of interest and the primary outcome for a future trial. In particular, whether we should focus on anxiety and depression or emotional regulation and self‐esteem is a question that will be clarified through further work with our stakeholders. Finally, the wider context of uncertainty and changing restrictions related to the ongoing COVID‐19 pandemic likely contributed considerably to the variability in our outcomes.

At baseline, our participants' average score on anxiety and depression symptoms was below clinical range, and similar to comparable South African samples (Visagie, Loxton, Swartz, & Stallard, 2021) but higher than samples recruited in schools in the United Kingdom ((Stallard et al., 2014)—for example mean total RCADS‐30 = 12.89). This indicates, as we anticipated, that in this LMIC context and peri‐pandemic, where vulnerability factors are high, many young adolescents may be experiencing some anxiety and depression symptoms, demonstrating the need for mental health promotion interventions.

When we designed 4STMF and this current study, we had not anticipated the global pandemic. It is very encouraging that we were able to deliver the 4STMF programme in schools even in the context of ongoing COVID‐19 restrictions; during the trial, physical distancing had to be maintained (which meant children coming to school every other day) and everyone had to wear face masks covering their mouth and nose. This is particularly salient given that our qualitative work in the first year of the pandemic in the Western Cape had further highlighted the great need for young adolescents to have a space to talk about their emotions and strategies to help them to manage these (Coetzee, Gericke, Human, Stallard, & Loades, 2021). One key factor which we believe aided our successful delivery of the programme was our collaboration with an NGO, Community Keepers (CK), who were already actively providing psychosocial support to learners in both schools. Other work has also found that it is more likely for mental health interventions to be successfully implemented at schools that partner with mental health organizations (Langley, Nadeem, Kataoka, Stein, & Jaycox, 2010). A particular benefit of having CK as a research partner was the safety net this provided, with the ability for our facilitators and research team to refer any participants who needed additional emotional support. During this study, 3 learners were referred to CK.

Delivering school‐based prevention programmes in the South African context for young adolescents is very different to that of high‐income countries (HICs). One key point of difference is that the comparator in those trials, that is curriculum as usual, looks very different and includes a greater emphasis on health and well‐being than in South Africa. Another key point of difference is the universality of mental health risk factors such as exposure to trauma and violence in the South African context (Hiller et al., 2017). Also, compared to schools in HICs, our schools (which receive food support from feeding schemes) have considerably fewer resources in terms of technology and time. Time constraints for intervention delivery were particularly salient in our second school when intervention delivery took place close to exam time. We specifically developed 4STMF based on detailed stakeholder work (Coetzee, Gericke, Human, Stallard, & Loades, 2022; Human, Gericke, Loades, Stallard, & Coetzee, 2022), which endorsed the need for this sort of programme. Based on the work reported here, we have found that 4STMF is both feasible and acceptable. To know whether it works, we need a fully powered trial across sites, powered for subgroup analyses including those with elevated symptoms at baseline, thus shedding light on what works for whom.

### Strengths and limitations

The 4STMF programme was developed in the local context with stakeholders, meaning that session length and delivery could be accommodated within school timetables thereby facilitating wide adoption, leading to longevity and sustainability. We measured a wide range of outcomes and showed the feasibility and acceptability of a pragmatic, implementation focused and flexibly adapted delivery according to needs and context. A key facilitator for engaging schools and then managing risk issues where they arose was having a collaborative relationship with the NGO who provided more targeted help within the schools who participated. Further work will be needed to determine how 4STMF can be scaled up beyond the schools in this NGO's network.

In terms of limitations, this was a feasibility study, and not a fully powered trial, and as such we only included two schools. Although selected at random, we are unsure how representative these schools are of the wider population.

Secondly, although the measures we selected are psychometrically validated, they have not been locally validated in this context of use (Western Cape in South Africa, peri‐pandemic). Several of our measures showed poor internal consistency at baseline, indicating that more work on the reliability of the measures in this context may be needed. Furthermore, it may be that we missed important nuances of how symptoms and experiences present and are reported in this cultural context, and further work is needed to ensure that measures used capture the range of experiences in a meaningful way.

Thirdly, at school 2, only 64% of participants completed one‐month follow‐up measures, which is considerably lower than retention rates at school 1. This may be because follow‐up data collection at school 2 coincided with the last week of the school term, just after the learners finished their end of term tests and learner absenteeism rates were higher than usual. Timings of measurement points in relation to key academic assessments may be useful to consider carefully in planning trials within school contexts.

Finally, we only followed participants up for one month postintervention. Arguably, for a preventative intervention and to be confident of longer‐term impacts, much longer follow‐up periods are needed.

## Conclusion

Despite these limitations, this pilot study has shown that the delivery of a bespoke, locally developed school‐based mental health programme for children in South Africa is feasible. A universal intervention is a pragmatic option for LMICs with limited mental health resources. Furthermore, the intervention can be delivered by nonmental health specialists with limited training, is brief and can be flexibly fitted within busy school timetables. Although feasible, further work is now required in a fully powered RCT to evaluate the effectiveness of 4STMF.

## Ethical information

All participants provided written informed consent and assent for participation in this study. Parental opt‐out consent was used for programme delivery. Parental consent was obtained for those participants who took part in the focus groups. The authors received permission to conduct this study from the Social, Behavioural and Education Research Ethics Committee (REC: SBER) at Stellenbosch University (project ID 9183) and reciprocity from the Psychology Research Ethics Committee, University of Bath. The authors also received permission from the Western Cape Department of Education.

## Supporting information


Appendix S1
