# Supplementary material for: Four Steps To My Future (4STMF): acceptability, feasibility and exploratory outcomes of a universal school‐based mental health and well‐being programme, delivered to young adolescents in South Africa
Source: Child Adolesc Ment Health. 2023 Jul 13;29(1):22–32. doi: 10.1111/camh.12660 (PMC10953368; doi:10.1111/camh.12660)
Supplement: Supplementary file 1 — Appendix S1 [file CAMH-29-22-s001.docx]

**Supporting Information**

**Table S1. themes and subthemes with example quotes**

| **Theme 1: Likeability of the programme** | **Example quote** |
| --- | --- |
| **Facilitator aspects:** Most learners spoke highly of their facilitators and reported that the facilitators were kind, friendly, good, helpful and made them feel better. Learners also said that the facilitators made the programme fun and allowed them to feel safe to express their emotions. In one instance a learner felt forced to take part in an activity. Learners wanted to be even more involved in discussions, and suggested that facilitators could engage them further by making a joke on occasion. | *it was nice/fun/good and she helped me with some of the things I struggled with (C1).* |
| **Programme aspects:** Learners enjoyed standing up and stretching between activities and learning about what to do when someone bullies you. Learners also said the programme helped them feel better, think about their future and was fun. While most enjoyed activities like the breathing activity and the kindness activity, some mentioned feeling shy doing these in class. Learners suggested that the programme could be further improved by including more games, limiting the number of worksheets, and by having more sessions. | *Yes it was a bit difficult for me to figure out how I actually feel or look when I’m upset (C2).*  *That was for me (the drawing activity) also because most of the time when I’m upset I won’t be close to a mirror and see how I look like I just had to think out of my head what expression was I making what type of face that I pull (C3).* |
| **Teacher presence:** Most learners said that they preferred it if their teacher was not present as they felt more reluctant to share their feelings in front of them, although others said that having the teacher present ensured better discipline. | *I think it would be better if the teacher is not in the class (C3).*  *I didn’t actually need the teachers in the class (C1).* |
| **Theme 2: Recall of the programme content:** When asked what they remembered about the programme, many learners talked about the 'catch-it, check-it, change-it' activity to identify, check and change negative thoughts. Others remembered the booklet they used to note down their helpful and unhelpful thoughts. Learners also recalled the drawing activity about feelings and how these may be expressed in the body, and the wristbands they received. They also remembered that there were 4 steps to the programme, giving compliments to others and being kind and the classroom posters. | *I remember a girl, she heard her friends talking about her other friends (who) went to eat ice-cream but they always invite her and then she didn’t feel good Miss so I said we can- so her teacher said we have to catch it, check it out and change it (C1).* |
| **Theme 3: Skills use in everyday life:** Most participants said that they continued to use the 4, 5, 6 breathing technique after the programme ended. Others reported giving compliments to others, using the stop and go thoughts booklet, the catch-it, check-it, change-it activity and believing in themselves. Participants said that the 4 posters created for each step that were put up in their classrooms were helpful reminders of these skills. | *[you inhale] for five seconds one two three four five keep in for three (sucks in breath) and breathe out for three seconds again (C3).*  *…and then you make yourself feel comfortable at your desk and you think of a place where you feel safe (C2).* |
| **Theme 4: Sharing the programme with others:** |  |
| **Telling others about the programme:** When asked who they had told about 4STMF, learners mentioned telling their parents, siblings, and friends. Some learners specifically mentioned giving their parents the parent handouts at the end of each step. | *Yes I told my mother what we did during the day and give her the little handouts and then she reads it (C2).* |
| **Involving others in the programme:** When asked whether they thought the programme should be delivered to anyone else they knew, learners mentioned family members like their parents, siblings, and cousins. Learners suggested that the programme could also be delivered by others like themselves, their parents, or family members. | *…miss I can deliver the programme to my parents myself. […] because I think it would give them a good life (C4).* |

**Table S2. Pre-, post- and one month within group differences at School 1**

|  | **School 1**  **(n=97)** | | | **Pre vs post** | **Pre vs 1 month** | **Post vs 1 month** |
| --- | --- | --- | --- | --- | --- | --- |
| **Scale** | **Pre-intervention, mean (SD), n** | **Post-intervention score, M (SD), n** | **1 month follow-up, M (SD), n** | **P & effect size** | **P & effect size** | **P & effect size** |
| Revised Child Anxiety and Depression Scale-30 | 32.69 (15.98) n=96 | 31.42 (16.37) n=89 | 28.47 (17.19) n=89 | 0.32,ES=0.07  (negl.) | <0.01,ES=0.26  (small) | <0.01,ES=0.20  (small) |
| Rosenberg self-esteem scale | 21.71 (4.53) n=97 | 28.32 (5.08) n=90 | 28.36 (5.73) n=89 | **<0.01, ES=1.36**  **(large)** | **<0.01, ES=1.37**  **(large)** | 0.95, ES=0.01 (negl.) |
| Emotion regulation questionnaire-cognitive reappraisal facet (CRF) | 20.38 (4.70) n=97 | 19.84 (4.80) n=90 | 18.68 (5.02) n=88 | 0.31,ES=0.11  (negl.) | <0.01,ES=0.35  (small) | 0.02,ES=0.24  (small) |
| Emotion regulation questionnaire-expressive suppression facet (ESF) | 12.94 (3.56) n=97 | 14.56 (3.84) n=89 | 12.13 (3.81) n=89 | **<0.01, ES=0.44**  **(medium)** | **0.03, ES=0.22**  **(small)** | **<0.01, ES=0.63**  **(medium)** |
| Olweus bullying questionnaire - Been bullied | 1.75 (0.98) n=95 | 1.83 (1.14) n=90 | 1.74 (0.97) n=89 | 0.37, ES=0.08  (negl.) | 0.76, ES=0.01  (neg.) | 0.57, ES=0.09  (negl.) |
| Olweus bullying questionnaire - Have bullied | 1.4 (0.78) n=94 | 1.53 (0.90) n=90 | 1.44 (0.74) n=89 | 0.22, ES=0.14  (negl.) | 0.62, ES=0.03  (negl.) | 0.47, ES=0.12  (negl.) |
| Goal-setting scale | 17.33 (3.66) n=96 | 18.27 (3.63) n=85 | 17.84 (3.64) n=86 | 0.08, ES=0.26  (small) | 0.38, ES=0.14  (negl.) | 0.39, ES=0.12  (negl.) |
| Happiness and well-being | 12.72 (5.62) n=97 | 13.29 (5.83) n=89 | 13.61 (6.40) n=89 | 0.1, ES=0.1  (negl.) | 0.04, ES=0.15  (small) | 0.64, ES=0.05  (negl.) |

Note: Negl. = negligible; ES = effect size (Cohen’s *d*); P = p value significance .0.5, two-tailed

**Table S3. Pre-, post- and one month within group differences at school 2**

|  | **School 2**  **(n=125)** | | | **Pre vs post** | **Pre vs 1 month** | **Post vs 1 month** |
| --- | --- | --- | --- | --- | --- | --- |
| **Scale** | **Pre-intervention, mean (SD), n** | **Post-intervention score, M (SD), n** | **1 month follow-up, M (SD), n** | **P & effect size** | **P & effect size** | **P & effect size** |
| Revised Child Anxiety and Depression Scale-30 | 28.30 (16.09) n=90 | 27.39 (15.31) n=98 | 27.86 (16.21) n=72 | 0.54, ES=0.06  (negl.) | 0.37,  ES=0.03  (negl.) | 0.72, ES=0.03  (negl.) |
| Rosenberg self-esteem scale | 27.29 (4.78) n=94 | 26.86 (4.56) n=105 | 27.22 (4.31) n=79 | 0.47 ES=0.09  (negl.) | 0.78 ES=0.02  (negl.) | 0.69 ES=0.08  (negl.) |
| Emotion regulation questionnaire-cognitive reappraisal facet (CRF) | 18.17 (5.19) n=94 | 17.94 (4.94) n=105 | 17.47 (5.22) n=79 | 0.45 ES=0.04  (negl.) | 0.03 ES=0.13  (negl.) | 0.12 ES=0.09  (negl.) |
| Emotion regulation questionnaire-expressive suppression facet (ESF) | 13.59 (4.03) n=94 | 11.40 (3.63) n=106 | 10.96 (3.31) n=79 | **<0.01 ES=0.57**  **(medium)** | **<0.01 ES=0.71**  **(medium)** | 0.34 ES=0.12  (negl.) |
| Olweus bullying questionnaire - Been bullied | 1.87 (1.23) n=122 | 1.88 (1.13) n=104 | 1.68 (1.00) n=78 | 0.81 ES=0.01  (negl.) | 0.12 ES=0.17  (small) | 0.09 ES=0.18  (small) |
| Olweus bullying questionnaire - Have bullied | 1.46 (0.81) n=122 | 1.69 (1.02) n=104 | 1.56 (0.89) n=78 | <0.01 ES=0.25  (small) | 0.30 ES=0.12  (negl.) | 0.21 ES=0.13  (negl.) |
| Goal-setting scale | 17.28 (3.74) n=93 | 16.54 (4.72) n=101 | 16.61 (3.58) n=77 | 0.11 ES=0.17  (small) | 0.16 ES=0.18  (small) | 0.94 ES=0.02  (negl.) |
| Happiness and well-being | 11.53 (5.37) n=94 | 11.58 (4.41) n=105 | 11.70 (5.53) n=79 | 0.85 ES=0.01  (negl.) | 0.78 ES=0.03  (negl.) | 0.91 ES=0.02  (negl.) |

Note: Negl. = negligible; ES = effect size (Cohen’s *d*); P = p value significance .0.5, two-tailed
